# Temporal distribution and genetic variants in influenza A(H1N1)pdm09 virus circulating in Mexico, seasons 2012 and 2013

**DOI:** 10.1371/journal.pone.0189363

**Published:** 2017-12-08

**Authors:** Jose Reyes Canche-Pech, Laura Conde-Ferraez, Marylin Puerto-Solis, Refugio Gonzalez-Losa, Pilar Granja-Pérez, Salha Villanueva-Jorge, Maria Chan-Gasca, Jesus Gómez-Carballo, Luisa López-Ochoa, Bertha Jiménez-Delgadillo, Iram Rodríguez-Sánchez, Jorge Ramírez-Prado, Guadalupe Ayora-Talavera

**Affiliations:** 1 Universidad Autonoma de Yucatan. Centro de Investigaciones Regionales Dr.Hideyo Noguchi. Av. Centro. C.P. Merida, Yucatan, Mexico; 2 Laboratorio Estatal de Salud Publica. Servicios de Salud de Yucatan, Yucatan, México; 3 Unidad de Bioquimica y Biologia Molecular de Plantas, Centro de Investigacion Cientifica de Yucatan, A.C., Calle, Col. Chuburna de Hidalgo, C.P. Merida, Yucatan, Mexico; 4 Universidad Autonoma de Yucatan. Facultad de Medicina. Av. Centro. C.P. Merida, Yucatan, Mexico; 5 Departamento de Genética, Facultad de Medicina, Universidad Autonoma de Nuevo Leon. Av. Gonzalitos s/n cruce con Av. Madero. Col. Mitras Centro. C.P. Monterrey, Nuevo Leon, Mexico; 6 Unidad de Biotecnologia, Centro de. Merida, Yucatan, Mexico; Pfizer Inc, UNITED STATES

## Abstract

The 2012 and 2013 annual influenza epidemics in Mexico were characterized by presenting different seasonal patterns. In 2012 the A(H1N1)pdm09 virus caused a high incidence of influenza infections after a two-year period of low circulation; whereas the 2013 epidemic presented circulation of the A(H1N1)pdm09 virus throughout the year. We have characterized the molecular composition of the Hemagglutinin (HA) and Neuraminidase (NA) genes of the A(H1N1)pdm09 virus from both epidemic seasons, emphasizing the genetic characteristics of viruses isolated from Yucatan in Southern Mexico. The molecular analysis of viruses from the 2012 revealed that all viruses from Mexico were predominantly grouped in clade 7. Strikingly, the molecular characterization of viruses from 2013 revealed that viruses circulating in Yucatan were genetically different to viruses from other regions of Mexico. In fact, we identified the occurrence of two genetic variants containing relevant mutations at both the HA and NA surface antigens. There was a difference on the temporal circulation of each genetic variant, viruses containing the mutations HA-A141T / NA-N341S were detected in May, June and July; whereas viruses containing the mutations HA-S162I / NA-L206S circulated in August and September. We discuss the significance of these novel genetic changes.

## Introduction

Mexico and the United States of America were the first two countries to report the occurrence of the novel A(H1N1)pdm09 virus in April 2009 [1, 2]. Since then and in the following years, the A(H1N1)pdm09 virus has been associated to annual epidemics and it is actually identified as a seasonal virus together with influenza A(H3N2) and influenza type B virus.

The epidemiological characteristics of the influenza A(H1N1)pdm09 virus in Mexico has been extensively described during different seasons, since the initial wave of the pandemic in 2009 to the further recurrence of the A(H1N1)pdm09 virus in season 2011–2012 and 2013–2014 [3–6]. Likewise, some studies have specifically reported the epidemiology in different geographic regions [7–9]. Under the same framework, the molecular characterization of the A(H1N1)pdm09 virus is also a key component of the surveillance system worldwide. Conversely to the epidemiological description, in Mexico, there exists an enormous gap of knowledge about the molecular evolution of the influenza A(H1N1)pdm09 virus further after the initial characterization at the time of the virus emergence [10, 11]. Differences in seasonal patterns of circulation [3] as well as geographic differences between the 32 Mexican states may influence the evolution of the A(H1N1)pdm09 virus as has been described in other regions of the world [12–14]. The molecular characterization of the A(H1N1)pdm09 virus in Mexico has been limited to studies that analysed a reduced number of viral isolates from the influenza season 2011–2012 [15], or to studies that analysed clinical samples by deep sequencing to search for viral markers of pathogenicity associated with severity and mortality [16, 17].

In the present study we describe the sequence analysis of hemagglutinin (HA) and neuraminidase (NA) from Mexican A(H1N1)pdm09 viruses submitted during 2012–2013 to the public databases. The molecular analysis also included 98 HA and NA sequences of viruses isolated in Yucatan during the recurrence of the A(H1N1)pdm09 virus in 2012 and the following year 2013. The molecular characterization of all the sequences for the year 2013 identified the presence of two genetic variants circulating exclusively in Yucatan. The phylogenetic analysis clustered these variants as independent monophyletic groups, suggesting the occurrence of natural events of genetic evolution. These results underline the need to close monitor the evolution of the influenza virus through the molecular analysis in countries as vast as Mexico.

## Materials and methods

### Clinical material and viral isolation

Clinical material (throat swabs) was obtained from the repository of the Health Ministry Regional Laboratory in Yucatan. Samples had been collected during the influenza season 2012 and 2013 as part of the sentinel surveillance system. Prior to storage at -70 °C, all samples were confirmed as positive to the A(H1N1)pdm09 virus by real time RT-PCR following CDC protocol [18]. The Health Ministry Laboratory provided a list of all tested samples for each influenza season (2012 and 2013). No epidemiological, demographic or personal data were proportioned with the clinical sample, only the identification number (ID) assigned during reception.

Viral isolation was performed in Madin Darby Canine Kidney cells (MDCK). The MDCK cells were seeded in 24-well plates and overnight incubated at 37 °C in a CO_2_ incubator with 5% of CO_2_. An aliquot of each clinical sample (200 μl) was inoculated in duplicate and completed to a final volume of 500 μl per well with 1X Dubelco´s Modified Eagles Media (D-MEM- GIBCO) to avoid dryness during incubation. Plates were centrifuged at 2,500 rpm during 1 hour at 25 °C, the viral inoculum was removed and cells were incubated with 1 ml of 1X D-MEM supplemented with TPCK- trypsin (SIGMA) at a final concentration of 2 μg/ml. Infected cells were monitored daily until cytopathic effect (CPE) and monolayer destruction was observed. Supernatant was harvested, centrifuged at 2,500 rpm/10 minutes to remove cell debris, aliquot and stored at—70 °C until further use. Previous to storage, the presence of the A(H1N1)pdm09 virus in the supernatant was detected by a conventional hemagglutination (HA) assay using a 1% suspension in PBS of turkey red blood cells, and confirmed by real time RT-PCR.

### Real-Time RT-PCR from viral isolates to confirm the A(H1N1)pdm09 virus

All viral isolates with an HA titre were confirmed as A(H1N1)pdm09 virus by real-time RT-PCR according to CDC protocol [18]. The sequence of primers and FAM-labelled TaqMan probes were actualized according to the InDRE protocol [19]. Previous to confirmation, viral RNA was extracted by automated protocols using the MagnaPure LC 2.0 Instrument and the MagnaPure LC total nucleic acid isolation kit protocol (Roche). The amplification procedure was performed with SuperScript III RT/Platinum Taq Mix Kit (Invitrogen) under the following reaction conditions, 5 μl of viral RNA, 1X Reaction Mix, 1 unit of RT/Platinum Taq enzyme mix, 800 nM of each primer, 200 nM of TaqMan probe, and molecular grade water for a final volume of 25 μl. A negative and a positive control were included for each run. The amplification conditions were, for the RT step: 1 cycle at 50 °C for 15 min and 1 cycle at 95 °C for 5 min; followed of 40 cycles at 95 °C for 15 sec and 1 min at 60 °C. All real-time RT-PCR amplifications were performed using a StepOnePlus real-time PCR system (Applied Biosystem), and data analysis using the OneStep Software V3.

### RT-PCR amplification of HA and NA genes

HA and NA full gene amplification was performed by conventional RT-PCR according to CDC protocol [20]. A set of ten or 8 primers (**Table 1**) were used to amplify five or four overlapping fragments covering the complete HA and NA genes, respectively. Amplification procedures were performed with the SuperScript One Step RT-PCR with platinum Taq kit (Invitrogen) under the following reaction conditions: 1X of Mastermix, 400 nM of each primer, 1 μl of the enzyme mix, 2 μL of viral RNA and molecular grade water to complete a final volume of 25 μl. Amplification conditions for the RT step were 1 cycle at 50 °C for 30 min and 1 cycle at 94 °C for 2 min, followed of 30 cycles at 94 °C for 20 sec, 50°C for 30 sec and 72 °C for 1 min, with a final extension step at 72 °C for 7 min. All amplifications were performed in a C-1000 Thermal cycler (BIO-RAD). PCR products were visualized in 1% TAE-Agarose gels stained with Sybr Safe (Invitrogen).

**Table 1:** Sequence of primers used to amplify the full HA and NA genes of the influenza A(H1N1)pdm09 virus.

Target	Fragment	Primer sequence
HA	1	HA1F- TGTAAAACGACGGCCAGTATACGACTAGCAAAAGCAGGGG
		HA461R- CAGGAAACAGCTATGACCTCATGATTGGGCCAYGA
	2	HA351F- TGTAAAACGACGGCCAGTACRTGTTACCCWGGRGATTTCA
		HA943R- CAGGAAACAGCTATGACCGAAAKGGGAGRCTGGTGTTTA
	3	HA379F- TGTAAAACGACGGCCAGTACRTGTTACCCAGGRGATTTC
		HA1204R- CAGGAAACAGCTATGACCTCTTTACCYACTRCTGTGAA
	4	HA1124F- TGTAAAACGACGGCCAGTTGGATGGTAYGGTTAYCAYCA
		HA1541R- CAGGAAACAGCTATGACCTCATAAGTYCCATTTYTGA
	5	HA1204F- TGTAAAACGACGGCCAGTAAGATGAAYACRCARTTCACAG
		HA1778R- CAGGAAACAGCTATGACCGTGTCAGTAGAAACAAGGGTGTTT
**NA**	1	NA0F- TGTAAAACGACGGCCAGTAGCAAAAGCAGGAGT
		NA600R- CAGGAAACAGCTATGACCCTGGACCRGAAATTCC
	2	NA536F- TGTAAAACGACGGCCAGTGGTCAGCAAGCGCATGYCATGA
		NA1346R- CAGGAAACAGCTATGACCGCTGCTYCCRCTAGTCCAGAT
	3	NA726F- TGTAAAACGACGGCCAGTAATGGRCARGCCTCRTACAA
		NA1346R- CAGGAAACAGCTATGACCGCTGCTYCCRCTAGTCCAGAT
	4	NA941F- TGTAAAACGACGGCCAGTTAGGATACATCTGCAGTGG
		NA1452R- CAGGAAACAGCTATGACCAGTAGAAACAAGGAG

### Sanger sequencing of HA and NA genes

Prior to sequencing, all PCR products were purified using the enzyme EXOSAP-IT (Invitrogen); each mixture containing 4 μl of Exosap-it and 2 μl of PCR product was incubated at 37 °C for 15 min followed of 80 °C during 15 min and immediately stored at 4 °C until use in the sequencing reaction. The sequencing reaction was performed in a final volume of 20 μl using the BigDye Terminator v1.1 Cycle sequencing kit (Applied Biosystems) under the following conditions: 4 μl of buffer, 2 μl of BigDye, 3.2 μM of M13 primers [20] and 1 μl of purified PCR product. Cycling conditions were as follow 1 cycle at 96 °C for 1 min and 25 cycles at 96 °C for 10 sec, 50 °C for 5 sec and 60 °C for 4 min. PCR products were purified using 96-well CentriSep plates (Applied Biosystems) to remove excess of primers and labelled ddNTPs. Purified products were concentrated and eluted in 20 μL of Hi-Di Formamide (Applied Biosystems), heated at 95°C for 5 min and immediately placed at 4 °C just before the run in a 310 genetic analyzer (Applied Biosystem) using a performance optimized polimer-6 and a 310GA 61cm x50 umid Capillary.

### Phylogenetic analysis

Sequence analysis of 98 HA and NA full length genes from A(H1N1)pdm09 viruses isolated from Yucatan during the 2012 and 2013 was performed with Geneious v6.0 (accession numbers are listed in **S1 Table**). All sequences were deposited at the GISAID influenza virus database (http://platform.gisaid.org). Additionally, we retrieve 61-HA and 46-NA sequences from Mexico (non-Yucatan) that were available from the two years of study; however, the sequence and phylogenetic analysis was performed only with full-length sequences (35-HA and 22-NA). The analysis also included 443-HA and 445-NA full length sequences from isolates collected world-wide taking randomly sequences representative of all the continents deposited during the two years of study. Sequences were retrieved from the GISAID and the NCBI public database. Phylogenetic analysis was done with Mega v5. Independent phylogenetic trees for HA and NA from each epidemic period were constructed using PHYML (maximum likelihood phylogenies), the HKY85 (Hasegawa-Kishino-Yano, 85) model with a SH-like branch support. All trees were constructed using the reference strain A/California/07/2009 as out-group.

## Results

### Temporal distribution of influenza A(H1N1)pdm09 virus during the period 2012 and 2013

#### Year 2012

The influenza season in Mexico had a recurrence of the A(H1N1)pdm09 virus starting in early December 2011 and continued until April 2012 [6, 15, 21]. Interesting, the recurrence of the A(H1N1)pdm09 virus arose after a previous season of undetectable levels of circulation except for the particular outbreak in the Chihuahua state in March 2011, where several associated deaths were reported [22]. From December 2011 to April 2012, the A(H1N1)pdm09 virus was wide-spread in the Mexican territory mainly concentrated in the central region, with low activity in the Southern states (**S1 Fig**). Particularly in Yucatan, the presence of the virus was reported until January 2012, with the highest peak of detection in February to decline in March (**S2 Fig**). According to the report from the Direccion General de Epidemiologia (DGE), the recurrence of the A(H1N1)pdm09 virus caused at the national level 6,021 cases confirmed by laboratory and Yucatan contributed only with 1.2% (76 cases). Clinical samples for viral isolation were selected according to the temporal occurrence in Yucatan. For the year 2012 we selected a total of 48 clinical samples, viral isolation in MDCK cells was achieved in 35 (73%) distributed during the three months of activity.

#### Year 2013

During the year 2013 the temporal distribution of the A(H1N1)pdm09 virus in Mexico was clearly different to the previous season. Overall, the A(H1N1)pdm09 virus had an extended season in Mexico starting from middle May 2013 until May 2014 [5, 23, 24]. The A(H1N1)pdm09 virus was widely distributed in the Mexican territory with a highest rate of detection than previous seasons; importantly the pattern of circulation showed a geographic shift from the Southern to the Central/Northern states. From May to December 2013 Yucatan contributed with a total of 144 confirmed cases (20% of positivity at national level). In particular according to the report by the Regional Laboratory, the A(H1N1)pdm09 virus was detected in Yucatan as early in February throughout the year with the highest peak in the summer months of June, July and August, at the same time that the influenza virus H3N2 (**S2 Fig and S3 Fig**); a drop in virus detection occurred after September with sporadic cases the rest of the year. To analyse the molecular evolution of the A(H1N1)pdm09 virus in Yucatan during this year, viral isolates were recovered from a total of 45 (31%) clinical samples distributed from May to October 2013.

### Molecular characterization of the hemagglutinin and neuraminidase—Season 2012

The molecular characterization of the A(H1N1)pdm09 virus for the year 2012 was performed in a total of 21 HA and 16 NA full gene sequences from Yucatan. The complete sequence of matching HA and NA genes was available in a total of 14 viruses (**S1 Table**). HA sequences from Yucatan showed 99% of homology within them, 99% homology against the other Mexican sequences analyzed in this study, and 98% homology compared to the reference strain A/California/07/2009.

#### HA

The molecular characterization compared to the reference strain identify for the HA a total of eleven amino acid changes (**Table 2**) detected in 95% of the sequences from Yucatan. Amino acid changes S185T, S203T, S143G and A197T at the HA1 region placed viruses from Yucatan at clade 7 in agreement with viruses isolated from Mexico (non-Yucatan) and around the world [25]. Interestingly, mutation D97N that grouped viruses in clade 6 was absent in all sequences available from Mexico and it was detected only in one sequence from Yucatan (A/Yucatan/86/2012), besides it was already in circulation in 55% of sequences from other regions of the world. Overall, all sequences from Mexico including viruses from Yucatan contain in addition two mutations S69T and N260D [15] that were detected in only 43% of viruses analysed in this study mainly in viruses from North America. Amino acid changes at the antigenic sites S185T and S203T were detected in all Mexican sequences.

**Table 2:** Amino acid changes and frequency of occurrence in the hemagglutinin (HA) protein of the influenza A(H1N1)pdm09 viruses isolated in Yucatan. Frequencies of detection for each mutation are also indicated for sequences from other regions of Mexico (non-Yucatan) and worldwide for the period 2012–2013. Percentages were calculated based on the total number of sequences analysed in this study (*n*). Empty cells indicate values = 0.

	Yucatan		Mexico		World	
**Mutation**	2012 (*n* = 21)	2013 (*n* = 26)	2012 (*n* = 18)	2013 (*n* = 7)	2012 (*n* = 178)	2013 (*n* = 265)
**S69T**	100	3.8	100		43	
**P83S**	100	96	94	100	97	100
**D97N**	4.8	96		100	55	91
**I116M**	4.8	38		14.3	<1	3
**A141T**	4.8	42			<1	2
**S143G**	100		100		53	8
**S162I**		46		14.3	1	
**K163Q**		8		86	3	50
**V173I**	10				4	4
**L174I**	10					
**S185T**	100	100	100	100	96	97
**A197T**	95		100		56	7
**S203T**	100	100	100	100	100	100
**R205K**		46		14.3	3	2
**V234I**		92			9	38
**A256T**		8		86	3	50
**N260D**	100		100		43	
**K283E**		100		100	14	88
**I321V**	100	100	100	71	95	94
**S359A**		8				
E47K*	100	92	100	100	100	99
S124N*	100	100	100	100	96	98
E172K*		92		100	22	94
V193A*	100	8	100		44	7

* Amino acid changes in HA2 region.

The phylogenetic analysis of the HA sequences grouped together the Yucatan viruses characterized by the presence of the mutation S69T, but separated from the other Mexican sequences included in the study (**S4 Fig**). The single virus with the HA mutation D97N was distant from the rest of the viruses in a single branch; it probably indicates the beginning of circulation of clade 6 viruses in Yucatan. According to the phylogeny, there was a close relationship between all Mexican viruses analysed in this study along with viruses from North America and Latin American countries. Noteworthy is the fact that the season 2012 in Mexico was caused by viruses representing the clade 7 grouped in a big branch separated from 2012 viruses from clade 6 that were already in circulation.

#### NA

The molecular analysis of the NA gene in A(H1N1)pdm09 viruses from Yucatan showed the presence of mutations N44S, V106I, V241I, N248D, and N369K commonly present in virus from other regions of Mexico and the world (**Table 3**). In accordance with the HA gene, all NA sequences from Mexico including Yucatan contained the mutation G41R reported for viruses grouped in clade 7, although the frequency of detection in viruses from other regions of the world was of 45%. The mutation H275Y associated to neuraminidase inhibitors resistance was detected in 10 sequences from Mexico but none from Yucatan. The percentage of similarity between the sequences from Yucatan and the reference strain was of 99% and within them of 100% identity.

**Table 3:** Amino acid changes and frequency of detection in the Neuraminidase (NA) protein of the influenza A(H1N1)pdm09 virus isolated from Yucatan, Mexico (non-Yucatan) and other regions of the world for the period 2012–2013. Percentages were calculated based on the total number of sequences analysed in this study (*n*). Empty cells indicate values = 0.

	Yucatan		Mexico		World	
Mutation	2012 (n = 16)	2013 (n = 35)	2012 (n = 15)	2013 (n = 7)	2012 (n = 110)	2013 (n = 335)
**I34V**		6		86		46
**G41R**	100		100		45	
**N44S**	100	100	100	100	74	95
**V106I**	100		100		73	5
**N200S**				86	26	94
**L206S**		29				
**V241I**	100	100	100	100	100	98
**N248D**	100	100	100	100	100	99
**I321V**		31		86	5.5	48
**N341S**		31				<1
**N369K**	100	100	100	100	100	98
**I393V**		11				
**N397K**				71		24
**K432E**		6		86		46
**D451G**		40		28.6	2.7	3

The phylogenetic analysis of NA sequences from Yucatan and Mexico showed a similar topology to the HA tree. Viruses from Yucatan grouped together but separated from viruses from other regions of Mexico, and viruses with the resistance mutation also grouped in a separate branch (**S4 Fig**). Viruses from Yucatan showed close relationship to viruses isolated from South and Central America as well as North America, suggesting that A(H1N1)pdm09 viruses circulating in Yucatan were probably introduced from these regions and not from viruses circulating in central/north Mexico.

### Genetic variants in viruses circulating in Yucatan during season 2013

The molecular characterization and phylogenetic analysis of the HA and NA sequences of viruses from Yucatan for the period 2013 showed the occurrence of two genetic variants that circulated at different times during the season. The percentage of homology within Yucatan sequences was of 99.8%, and compared to the reference strain A/California/7/2009 or the Mexican sequences from other regions the percentage of homology was of 98.3 and 99.1% respectively.

#### HA

The molecular characterization was performed in 26 full HA sequences from Yucatan and 7 from other regions of Mexico (available at the GISAID database). From all mutations detected in the previous year 2012, only six were conserved (P83S, S185T, S203T, I321V, E374K y S451N). The loss of mutations S143G and A197T and the occurrence of three new mutations, D97N, V234I and K283E at the HA1 region, place all A(H1N1)pdm09 viruses from Mexico including viruses from Yucatan in the predominant clade 6, subgroups 6B and/or 6C [25]; these mutations were accompanied by E47K and E172K at the HA2 region (**Table 1**). Interesting, we identified two mutations I116M and R205K at frequencies of 38% and 46% respectively in viruses from Yucatan and detected in only one virus from Mexico (non-Yucatan), and at very low frequencies of 3% in viruses from other regions of the world analysed in our study. These results suggest that viruses from subtropical Yucatan possessed particular genetic markers shared at very low frequencies with viruses globally in circulation.

**Fig 1:**
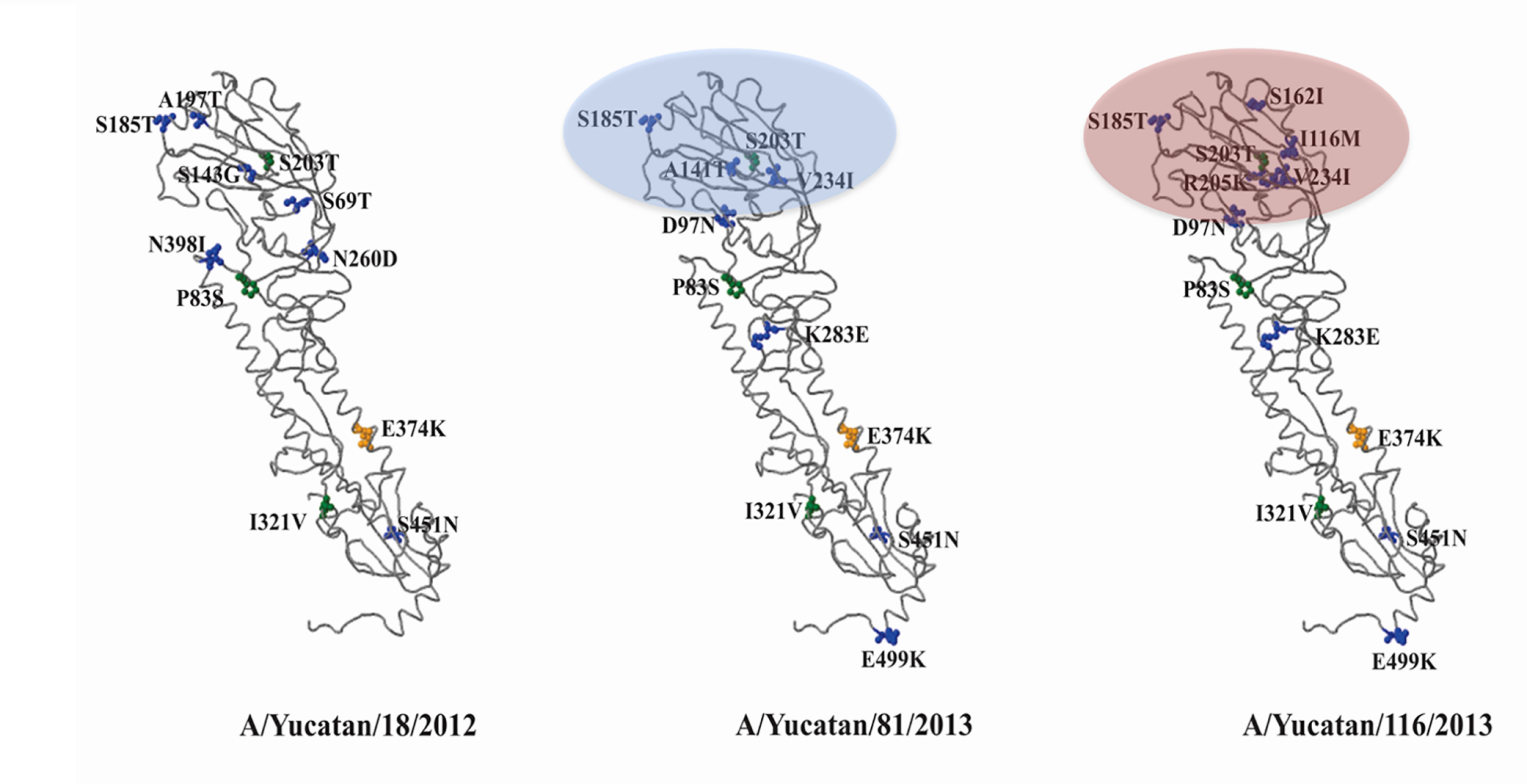
Schematic representation of the HA molecule of the A(H1N1)pdm09 virus. HA molecules were modelled using as template the crystal structure of A 2009 H1N1 virus hemagglutinin from A/California/04/2009 (PDB 3LZG). Molecules were generated under flusurver structural model database (http://flusurver.bii.a-star.edu.sg). Protein sequences were blast against A/California/07/2009 to identify differences on amino acid composition. Right side HA molecule was modelled based on the strain A/Yucatan/116/2013 to indicate localization of the amino acid change S162I (red oval). The HA molecule in the middle represents the amino acidic composition of the strain A/Yucatan/81/2013 indicating the position of the mutation A141T (blue oval). Mutations at residues V234I, K283E and E499K (HA2) placed these viruses in clade 6C. Left side molecule corresponds to the HA protein sequence of the strain A/Yucatan/18/2012.

The most significant finding in the A(H1N1)pdm09 viruses from Yucatan was the presence of two mutations A141T and S162I with percentages of frequency of 42% and 46% (**Table 1**); both mutations are localized at two antigenic sites at the HA globular head, Ca and Sa, respectively (**Fig 1).** Mutation A141T was present in only 2% of worldwide sequences analysed in this study with no presence in viruses from other regions of Mexico. Strikingly, mutation S162I was unique for sequences from Yucatan; it was identified in only one sequence from other regions of Mexico (A/Mexico/2733/2013) and in none of the sequences from other regions of the world analysed in this study.

**Fig 2:**
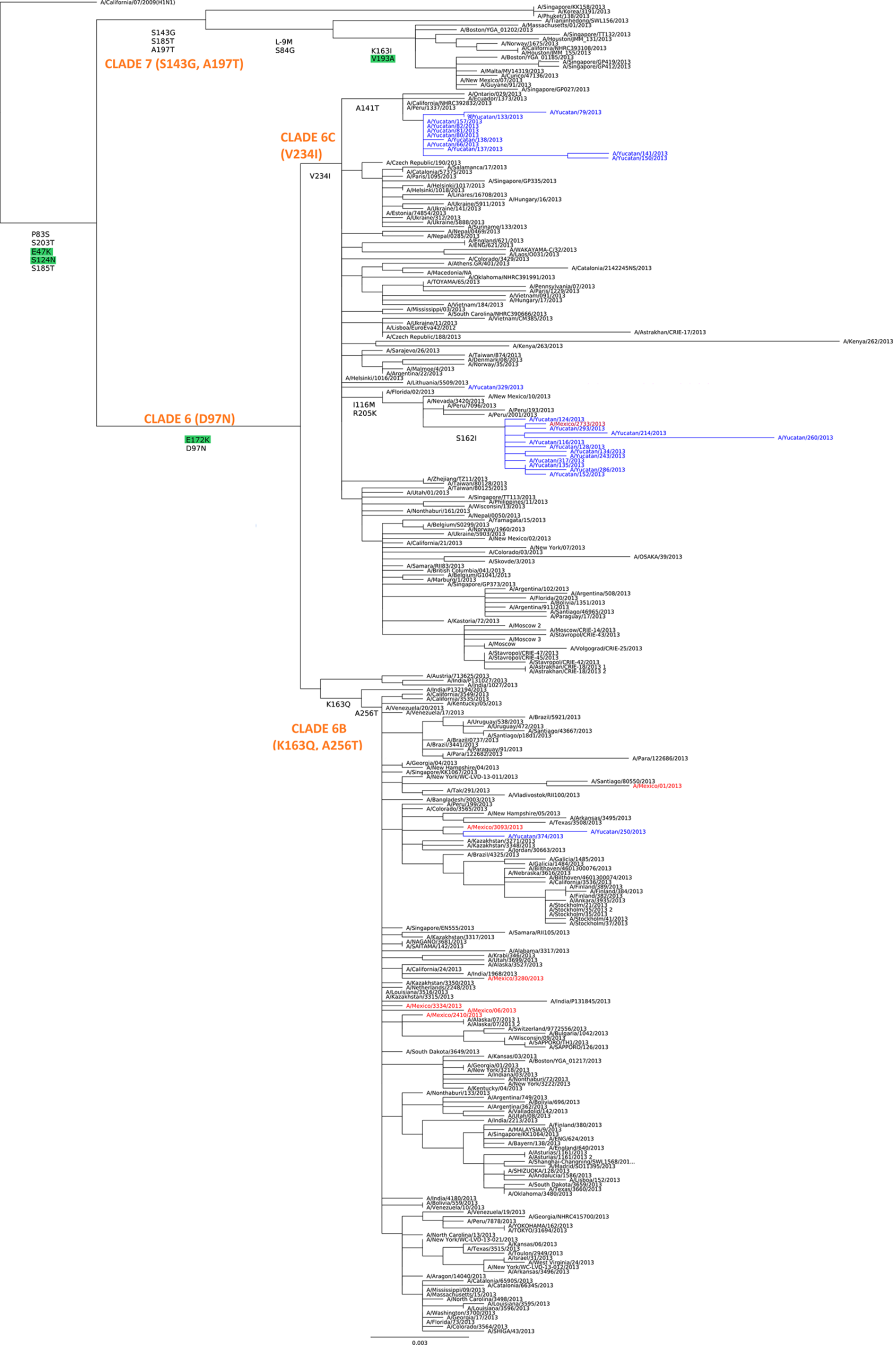
Phylogenetic tree of HA nucleotide sequences of A(H1N1)pdm09 viruses from the year 2013. Twenty-six HA sequences from Yucatan were aligned with 272 sequences from other regions of the world including seven from other regions of Mexico. Two independent clades were formed with sequences from Yucatan, corresponding to viruses containing either A141T or S162I amino acid mutation.

The phylogenetic analysis of the HA of viruses from the season 2013 clearly shows that viruses from Yucatan are grouped into three different clades, a group of 12 viruses containing the mutation S162I together with the virus from Mexico, a group of 11 viruses containing the mutation A141T, and two viruses grouping close to the other Mexican strains (**Fig 2**); no viruses containing both genetic changes were identified. Based on the classification by the OMS [25], viruses from Mexico (non-Yucatan) are grouped in clade 6B containing mutations D97N, K163Q, A256T and K283E. On the contrary, viruses from Yucatan are grouped in clade 6C containing mutations D97N, V234I and K283E. Viruses A/Yucatan/374/2013 and A/Yucatan/250/2013 were grouped in clade 6B along the other Mexican viruses; the virus A/Yucatan/329/2013 was grouped in clade 6C without the genetic changes we have described.

**Fig 3:**
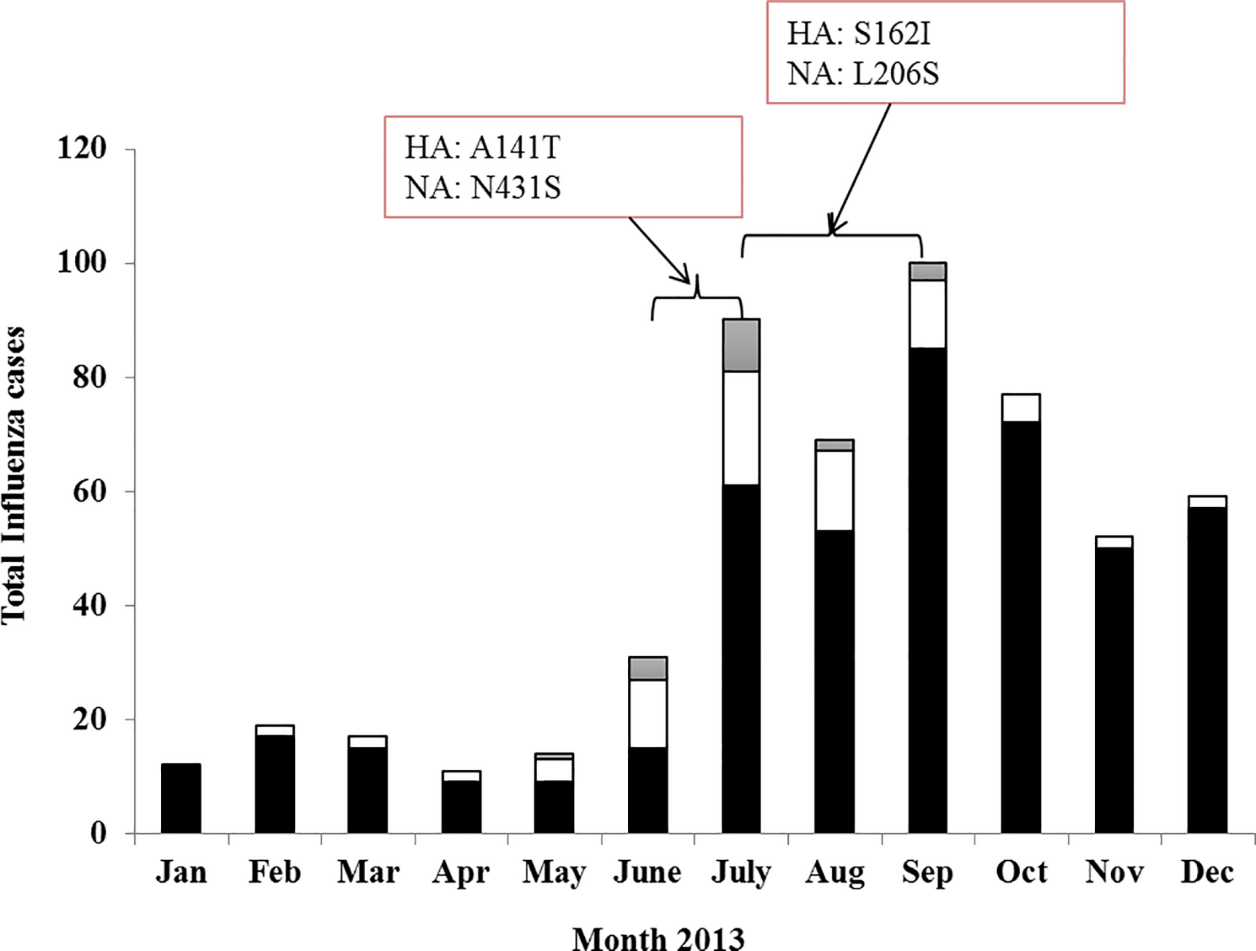
Temporal occurrence of genetic variants in Yucatan. Viruses from Yucatan with changes in HA and NA were distributed in time from epidemiological week 23 to 38. The bars represent the total number of influenza cases reported by the Regional Laboratory. The clear section indicates the number of confirmed cases of influenza A(H1N1)pdm09 and the grey section indicates the number of influenza A(H1N1)pdm09 viruses with genetic changes in HA and NA (HA–A141T / HA–S162I / NA–N341S / NA–L206S). The black section corresponds to the number of samples negative to influenza A(H1N1)pdm09 virus.

The temporal distribution of the A(H1N1)pdm09 virus during the season 2013 showed an extended period of circulation in Yucatan, with the highest frequency of circulation from June until September. The mutation A141T was detected in June in 25% (4 out of 16) of the A(H1N1)pdm09 viruses analysed, whereas the change S162I was detected in August (25%; 4 out of 16) and September (21%; 3 out of 14); co-circulation of viruses containing one or another genetic change occurred during the month of July (6 viruses with HA -A141T and 6 viruses with HA -S162I) (**S1 Table and Fig 3**).

#### NA

**Fig 4:**
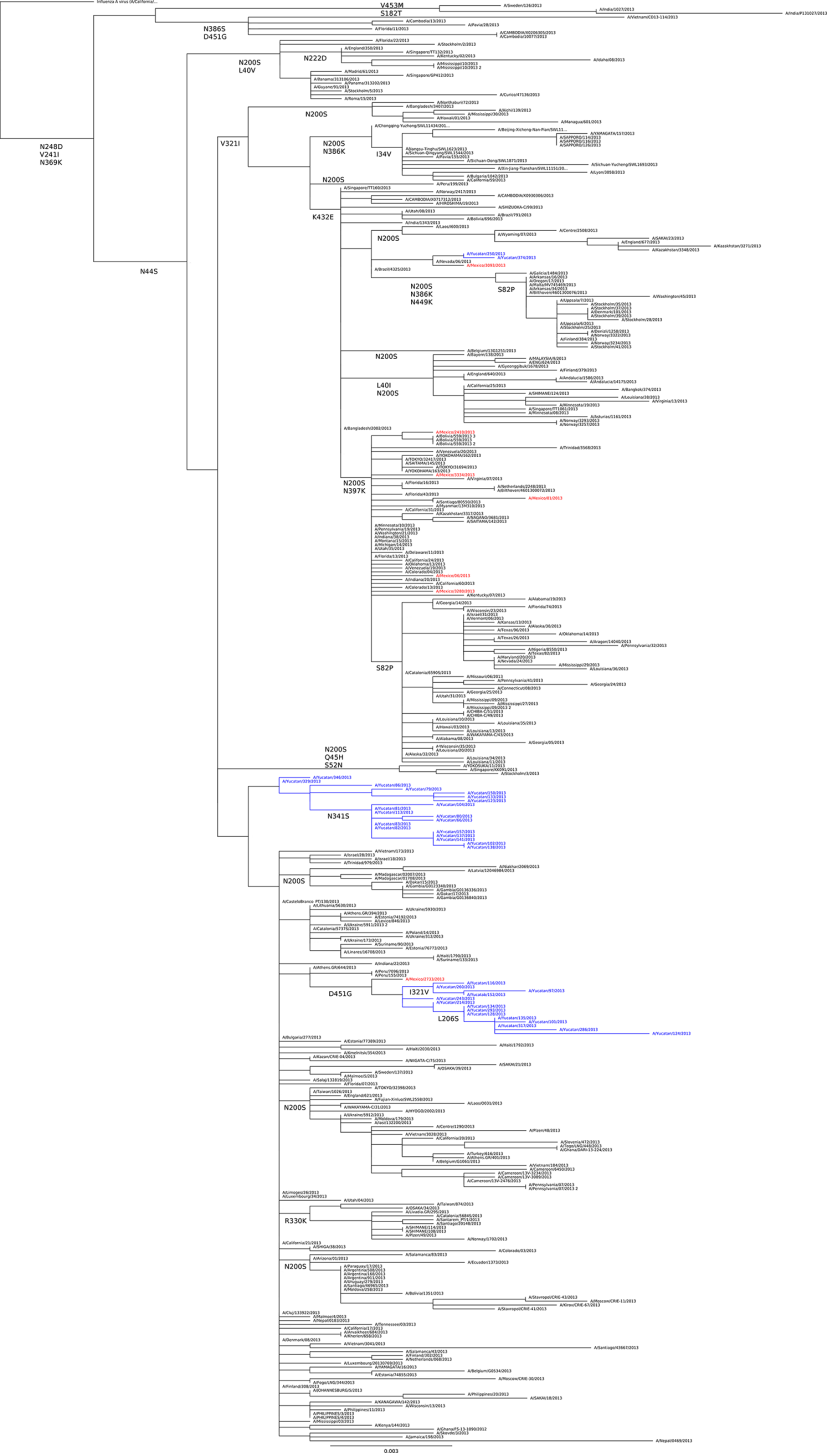
Phylogenetic tree of NA sequences of the A(H1N1)pdm09 viruses for the period 2013. Sequences from Yucatan are indicated in blue, from other regions of Mexico in red and worldwide in black. Additional amino acid changes at the node are also indicated.

The molecular analysis of the A(H1N1)pdm09 viruses isolated during the season 2013 was performed in a total of 35 full NA genes from Yucatan and compared with 7 NA sequences from other regions of Mexico. Four mutations were conserved in all viruses from Mexico and around the world, N44S, V241I, N248D and N369K (**Table 3**). The analysis identified viruses from Mexico (non-Yucatan) and worldwide still grouped in clade 6 and 7 by containing mutation N200S and the revertants R41G and I106V [25]. Interesting none of the viruses from Yucatan did acquire mutation N200S compared to sequences where the mutation was identified in 86% and 94% in viruses from other regions of Mexico and the world respectively. Another mutation that was absent in the Yucatan viruses was the N397K, which has been reported mainly in North America and it was present in 71% of sequences from other regions of Mexico analysed in this study. Moreover, we also identified four amino acid changes, L206S, N341S, I393V and D451G at variable frequencies in the Yucatan viruses but either absent or present in small numbers in viruses from other regions of Mexico and worldwide (**Table 3**). Mutations L206S and N341S were exclusive of the Yucatan viruses, the former mutation was identified in 8 NA sequences, whereas the last mutation was identified in 9 NA sequences and two viruses A/Yucatan/102/2013 and A/Yucatan/138/2013 contained both amino acid changes (**Fig 4**). The change I393V was identified in four NA sequences from Yucatan but was absent from all other sequences from Mexico and worldwide analysed in this study; opposite, in 2012, this mutation was identified in the NA sequences from Mexico (non-Yucatan) and the world, but not in the Yucatan viruses. The last mutation of relevance was the amino acid change D451G, which was found in a group of 14 sequences from Yucatan (40%), in one sequence from other regions of Mexico and in only 3% of sequences from the world.

The phylogenetic analysis of the NA sequences from Yucatan clustered viruses in six different groups according to the mutations we have previously described (**Fig 4**). The topology of the NA tree is similar to the HA (**Fig 2**), where the Yucatan viruses grouped separate from the viruses from other regions of Mexico. Two main clusters were formed, one for those viruses containing the mutation L206S but proceeded by the mutation D451G at the node; and the group of viruses containing the mutation N341S. These two mutations L206S and N341S in NA were detected in the same group of viruses where the HA genetic changes A141T and S162I were identified. A small subgroup within the D451G node was formed by viruses A/Yucatan/97/2013, A/Yucatan/116/2013, A/Yucatan/152/2013 and A/Yucatan/260/2013 characterized by lacking the mutation L206S, instead, they contained the mutation I321V which was highly prevalent in viruses from Mexico (86%) and worldwide (48%). Additionally, a second subgroup formed by viruses A/Yucatan/79/2013, A/Yucatan/123/2013, A/Yucatan/133/2013 and A/Yucatan/150/2013 was characterized by the amino acid change I393V although the N341S was absent within them. Finally, viruses A/Yucatan/250/2013 and A/Yucatan/374/2013 were characterized by grouping with viruses from other regions of Mexico containing mutations that were frequently detected worldwide (I34V and K432E); whereas viruses A/Yucatan/329/2013 and A/Yucatan/345/2013 characterized by lacking all of the mutation previously describe (**Fig 4**).

The analysis of the temporal distribution showed that the genetic change N341S was detected in 70% of A(H1N1)pdm09 viruses analysed in June (**S1 Table**). Then after, the genetic change L206S was detected in 50% of the A(H1N1)pdm09 viruses analysed from August and in 60% from September. The circulation of viruses with either genetic change was detected in July (N341S in 50%; L206S in 28.5%). These results clearly indicate the presence in Yucatan of a diverse pool of genetic variants of the influenza A(H1N1)pdm09 virus for the season 2013 (**Fig 3**).

## Discussion

The influenza A(H1N1)pdm09 virus resurged in the Mexican territory during the season December 2011- March 2012. The influenza season was characterized by a short period of circulation but with a high incidence after two years of very low detection [6]. The molecular characterization of a limited number of early isolates from the central region of the country revealed the presence of three dominant amino acid changes with respect to the vaccine reference strain A/California/07/2009 [15]. Further during the same season the genetic analysis of a larger number of HA and NA sequences including viruses from Subtropical Yucatan showed high genetic similitude between all the sequences from Mexico. The amino acid differences, S69T, S143G and N260D were detected in all the Mexican viruses analysed, and were also detected in viruses from North America particularly from the regions of California and Texas (**S4 Fig**). These amino acid changes seemed to represent a characteristic genetic marker of the season 2011–2012 [26]. It is of particular interest the high incidence of the A(H1N1)pdm09 virus during the season 2011–2012, even though the vaccination campaign in Mexico started a month before the peak of the season. Since the emergence in 2009, no immune scape variants have been identified for the A(H1N1)pdm09 virus that could change the HA antigenic profile, thus the vaccine strain composition for the H1N1 subtype has been maintain for the last seven years [27]. Therefore, the increased number of susceptible/naïve individuals after a long period of low transmission could directly have an impact in the number of infected population in 2012.

The molecular analysis of viruses isolated during the 2013 allowed us to identify the occurrence of genetic variants with amino acid changes in 27% of influenza A(H1N1)pdm09 viruses from Yucatan. Mutations detected in the HA -A141T and S162I- are located at the antigenic sites Ca_2_ and Sa respectively, and they were recently reported in the H1N1pdm09 virus as B-cell/Ab epitopes [28–30]. Influenza viruses can escape from the antibody-mediated neutralization by accumulating mutations in HA in order to evade the protective immunity. However, the evolution of the H1N1pdm09 virus has been remained antigenically homogeneous and similar to the vaccine strain A/California/07/2009-like [31–33].

The amino acid A141T (A144T in H3 numbering) is located in the antigenic site Ca_2_. This antigenic site together with Ca_1_ and Cb are considered induce cross-reactive protection. In H3 viruses this residue was associated with the emergency of escape mutants during the 1972/1975 epidemics and the occurrence of antigenically different strains (A/Memphis/72 to A/Victoria/75 [34, 35]. The relevance of this amino acid on (1) the antigenicity of the influenza virus, (2) the emergence of escape variants and (3) the response to neutralization, has been experimentally revealed in the presence of monoclonal antibodies against H3, H5 and recently the pandemic H1 virus [29, 36, 37]. Globally, mutation A141T has been reported in viruses from Canada with a frequency of detection of 30% in A(H1N1)pdm09 viruses isolated during the 2012–2013 season [38]. A critical second finding in our study was the detection of mutation N341S (N344S N2 numbering) in the NA of these viruses from Yucatan. This amino acid is located at the antigenic site 4 of the N1NA subtype [39] in proximity to the catalytic site, and has been implicated in the generation of antibody neutralization escape mutants in N2 and N8 viruses [40, 41].

The amino acid S162I (165 in H3 numbering) is located at the antigenic site Sa on the characterized H1HA [28]. This antigenic site induces an antibody response strain-specific, and very recently it has been described as experiencing “diversifying selection” [29]. Under this context, the evolutionary analysis of the A(H1N1)pdm09 virus since its emergency, indicated different events in this residue including the acquisition of a glycosylation site -S162N during the epidemic period 2010–2011 [25]; actually highly prevalent in more than 80% of the viruses circulating worldwide clustering viruses in a new sub-clade, the 6B.1 [42].

In this study we have described the emergence of genetic variants of influenza A(H1N1)pdm09 viruses in a specific region of Mexico. Overall, this study underlines the need of continuous monitoring of influenza viruses in different regions of Mexico with different seasonal patterns as we have recently shown [43], and the need to increase the number of sequences available to better characterize the influenza viruses circulating in larger countries as Mexico.

## Supporting information

S1 Table Accession number, virus name and date of collection of samples from Yucatan for the period 2012 and 2013. Viruses from 2013 were coloured in red to indicate the presence of matching HA/NA mutations A141T+N431S or blue to indicate the paired mutation S162I+L206S. Additionally, viruses with other mutations in NA were coloured in green for L206S+N341S; yellow I321V; pink I341V+K432E; or orange for viruses without any of the previous.
(XLSX)

S1 Fig Map of Mexico showing geographic regions: South and South-east (Yucatan, Campeche, Quintana Roo, Tabasco, Veracruz, Chiapas, Oaxaca, Guerrero); Central and Pacific Central (Puebla, Morelos, Distrito Federal, Hidalgo, Guanajuato, Jalisco, Colima, Nayarit, Aguascalientes, San Luis Potosí, Zacatecas); North-east and North-west (Tamaulipas, Nuevo León, Coahuila, Durango, Sinaloa, Chihuahua, Sonora, Baja California, Baja California Sur). (TIF)

S2 Fig Temporal distribution of influenza A(H1N1)pdm09 viruses detected during season 2012 and 2013. The black solid line represents number of confirmed cases by laboratory in Mexico including Yucatan (y axe). The dotted line represents number of confirmed cases by the Regional laboratory in Yucatan (secondary *y* axe).
(TIF)

S3 Fig Temporal circulation of influenza A and B in Yucatan during the 2013. Influenza AH3N2 and A(H1N1)pdm09 were circulating at the same time from May until October. The A(H1N1)pdm09 peaked in circulation from June to October whereas the H3N2 peaked from July to October. At the end of the year, the influenza epidemic was predominated by influenza B virus.
(TIF)

S4 Fig Phylogenetic tree of HA and NA genes of influenza A(H1N1)pdm09 virus isolated during 2012. Trees were constructed for HA and NA full-length nucleotide sequences from different regions of the world. Viruses are coloured in black to represent worldwide strains, red for viruses from Mexico (non-Yucatan), and blue for viruses specifically from Yucatan. Trees were constructed using the PHYML method with a SH-like branch support.
(TIFF)

## References

[1] CDC. Outbreak of Swine-Origin Influenza A (H1N1) Virus Infection—Mexico, March—April 2009. MMWR. 2009;58:467–70. 19444150

[2] CDC. Swine Influenza A (H1N1) Infection in Two Children—Southern California, March—April 2009. MMWR. 2009;58:400–2. 19390508

[3] Chowell G, Echeverría-Zuno S, Viboud C, Simonsen L, Tamerius J, Miller MA, et al Characterizing the epidemiology of the 2009 influenza A/H1N1 pandemic in Mexico. PLos Med. 2011;8:e1000436 doi: 10.1371/journal.pmed.1000436 21629683 10.1371/journal.pmed.1000436 PMC3101203

[4] Chowell G, Echeverría-Zuno S, Viboud C, Simonsen L, Miller MA, Fernández-Gárate I, et al Epidemiological characteristics and underlying risk factors for mortality during the Autumn 2009 pandemic wave in Mexico. PLoS ONE. 2012;7:e41069 doi: 10.1371/journal.pone.0041069 22815917 10.1371/journal.pone.0041069 PMC3397937

[5] Davila-Torres J, Chowell G, Borja-Aburto VH, Viboud C, Grajalez-Muñiz C, Miller MA. Intense seasonal A/H1N1 influenza in Mexico, winter 2013–2014. Arch Med Res. 2015;46:63–70. doi: 10.1016/j.arcmed.2014.11.005 25446618 10.1016/j.arcmed.2014.11.005

[6] Borja-Aburto V, Chowell G, Viboud C, Simonsen L, Miller MA, Grajales-Muñiz C, et al Epidemiological characterization of a fourth wave of pandemic A/H1N1 influenza in Mexico, winter 2011–2012: age shift and severity. Arch Med Res. 2012;43:563–70. doi: 10.1016/j.arcmed.2012.09.005 23079035 10.1016/j.arcmed.2012.09.005 PMC3545473

[7] Ayora-Talavera G, Betancourt-Cravioto M, Gomez-Carballo J, Conde-Ferraez L, Gonzalez-Losa R, Manrique-Saide P, et al Epidemiologic study of human influenza A(H1N1)pdm09 virus in Yucatan, Souther Mexico. Rev Biomed. 2012;23:39–46.

[8] Comas-Garcıa A, Garcia-Sepulveda CA, Mendez-de Lira JJ, Aranda-Romo S, Hernandez-Salinas AE, Noyola DE. Mortality attributable to pandemic influenza A(H1N1) 2009 in San Luis Potosi, Mexico. Influenza and Other Respiratory Viruses. 2011;5:76–82. doi: 10.1111/j.1750-2659.2010.00187.x 21306570 10.1111/j.1750-2659.2010.00187.x PMC4942002

[9] Gómez-Gómez A, Magaña-Aquino M, García-Sepúlveda CA, Ochoa-Pérez UR, Falcón-Escobedo R, Comas-García A, et al Severe pneumonia associated with pandemic (H1N1) 2009 outbreak, San Luis Potosi, Mexico. Emerg Infect Dis. 2010;16:27–34. doi: 10.3201/eid1601.090941 20031039 10.3201/eid1601.090941 PMC2874369

[10] Fereidouni S, Beer M, Vahlenkamp T, Starick E. Differentiation of two distinct clusters among currently circulating influenza A(H1N1)v viruses, March-September 2009. Euro Surveillance. 2009;14:19409 19941799

[11] Garten R, Davis CT, Russell CA, Shu B, Lindstrom S, Balish A, et al Antigenic and genetic characteristics of swine-origin 2009 A(H1N1) influenza viruses circulating in humans. Science. 2009;325:197–201. doi: 10.1126/science.1176225 19465683 10.1126/science.1176225 PMC3250984

[12] Nelson M, Tan Y, Ghedin E, Wenworth DE, George K, Edelman L, et al Phyleography of the spring and fall waves of the H1N1/09 pandemic influenza virus in the United States. J Virol. 2011;85:828–34. doi: 10.1128/JVI.01762-10 21068250 10.1128/JVI.01762-10 PMC3020026

[13] Mullick J, Cherian SS, Potdar VA, Chadha MS, Mishra AC. Evolutionary dynamics of the influenza A pandemic (H1N1) 2009 virus with emphasis on Indian isolates: Evidence for adaptive evolution in the HA gene. Infection, Genetics and Evolution. 2011;11:997–1005. doi: 10.1016/j.meegid.2011.03.015 21457796 10.1016/j.meegid.2011.03.015

[14] Fang Q, Gao Y, Chen M, Guo X, Yang X, Wei L. Molecular epidemiology and evolution of influenza A and B viruses during winter 2012–2014 in Beijing, China. Arch Virol. 2015;160:1083–95. doi: 10.1007/s00705-015-2362-x 25676826 10.1007/s00705-015-2362-x

[15] de la Rosa-Zamboni D, Vazquez-Perez JA, Avila-Rios S, Carranco-Arenas AP, Ormsby CE, Cummings CA, et al Molecular characterization of the predominant influenza A(H1N1)pdm09 virus in Mexico, December 2011-February 2012. PLoS ONE. 2012;7:e50116 doi: 10.1371/journal.pone.0050116 23209653 10.1371/journal.pone.0050116 PMC3510220

[16] Vazquez-Perez J, Isa P, Kobasa D, Ormsby CE, Ramírez-Gonzalez JE, Romero-Rodríguez DP, et al A(H1N1)pdm09 HA D222 variants associated with severity and mortality in patients during a second wave in Mexico. Virol J. 2013;10:41 doi: 10.1186/1743-422X-10-41 23369604 10.1186/1743-422X-10-41 PMC3583722

[17] Arellano-Llamas R, Alfaro-Ruiz L, Arriaga-Canon C, Imaz-Rosshandler I, Cruz-Lagunas A, Zúñiga J, et al Molecular features of influenza A(H1N1)pdm09 prevalent in Mexico during winter seasons 2012–2014. PLoS ONE. 2017;12:e0180419 doi: 10.1371/journal.pone.0180419 28692701 10.1371/journal.pone.0180419 PMC5503254

[18] WHO. CDC protocol of realtime RTPCR for influenza A (H1N1) 1999. 6 October 2009:[Available from: http://www.who.int/csr/resources/publications/swineflu/realtimeptpcr/en/.

[19] DGE. Lineamientos para la Vigilancia Epidemiológica de Influenza por Laboratorio.2015. Available from: http://portal.salud.gob.mx/archivos/lineamientos_para_la_vigilancia_de_influenza.pdf.

[20] WHO. Sequencing primers and protocol.2009. Available from: http://www.who.int/csr/resources/publications/swineflu/sequencing_primers/en/index.html.

[21] DGE. Informacion relevante de influenza. 2012 [September 2015]. Available from: http://www.epidemiologia.salud.gob.mx/informes/informesh/2012/influenza/influenza12_52.html.

[22] Houng H, Garner J, Zhou Y, Lyons A, Kuschner R, Deye G, et al Emergent 2009 influenza A(H1N1) viruses containing HA D222N mutation associated with severe clinical outcomes in the Americas. J Clin Virol. 2012;53:12–5. doi: 10.1016/j.jcv.2011.09.004 22036040 10.1016/j.jcv.2011.09.004

[23] DGE. Informacion relevante de influenza. 2013 [Accesed July 2015]. Available from: http://www.epidemiologia.salud.gob.mx/informes/informesh/2013/influenza/influenza13_52.html.

[24] DGE. Informacion relevante de influenza. 2014 [July 2015]. Available from: http://www.epidemiologia.salud.gob.mx/informes/informesh/2014/doctos/influenza/INFLUENZA_2014_SE20.pdf

[25] Klimov A, Garten R, Russell C, Barr IG, Besselarr TG, Daniels R, et al WHO recommendations for the viruses to be used in the 2012 Southern Hemisphere influenza vaccine: epidemiology, antigenic and genetic characteristics of influenza A(H1N1)pdm09, A(H3N2) and B influenza viruses collected from February to September 2011. Vaccine. 2012;30:6461–71. doi: 10.1016/j.vaccine.2012.07.089 22917957 10.1016/j.vaccine.2012.07.089 PMC6061925

[26] Klein E, Serohijos AWR, Choi JM, Shakhmovich EI, Pekosz A. Influenza A H1N1 pandemic strain evolution—divergency and the potential for antigenic drift variants. PLoS ONE. 2014;9:e93632 doi: 10.1371/journal.pone.0093632 24699432 10.1371/journal.pone.0093632 PMC3974778

[27] WHO. Recommended composition of influenza virus vaccines for use in the northern hemisphere 2016–2017 influenza season and development of candidate vaccine viruses for pandemic preparedness [Accessed 10 April 2016]. Available from: http://www.who.int/influenza/vaccines/virus/recommendations/201602_qanda_recommendation.pdf?ua=.

[28] Caton A, Brownlee GG, Yewdell JW, Gerhard W. The antigenic structure of the influenza virus A/PR/8/34 hemagglutinin (H1 subtype). Cell. 1982;31:417–27. 6186384 10.1016/0092-8674(82)90135-0

[29] Lee A, Das SR, Wang W, Fitzgerald T, Pickett BE, Aevermann BD, et al Diversifying selection analysis predicts antigenic evolution of 2009 pandemic H1N1 influenza A virus in humans. J Virol. 2015;89:5427–40. doi: 10.1128/JVI.03636-14 25741011 10.1128/JVI.03636-14 PMC4442545

[30] Squires R, Noronha J, Hunt V, García-Sastre A, Macken C, Baumgarth N, et al Influenza research database: an integrated bioinformatics resource for influenza research and surveillance. Influenza and Other Respiratory Viruses. 2012;6:404–16. doi: 10.1111/j.1750-2659.2011.00331.x 22260278 10.1111/j.1750-2659.2011.00331.x PMC3345175

[31] Su Y, Bahl J, Joseph U, Butt KM, Peck HA, Koay ESC, et al Phylodynamics of H1N1/2009 influenza reveals the transition from host adaptation to immune-driven selection. Nat Commun. 2015;6:7952–64. doi: 10.1038/ncomms8952 26245473 10.1038/ncomms8952 PMC4918339

[32] WHO. Recommended composition of influenza virus vaccines for use in the 2013–2014 northern hemisphere influenza season. 2013.

[33] WHO. Recommended composition of influenza virus vaccines for use in the 2014 southern hemisphere influenza season. 2013. 24159667

[34] Koel B, Burke DF, Bestebroer TM, van der Vliet S, Zondag GCM, Vervaet G, et al Substitutions near the receptor binding site determine major antigenic change during influenza virus evolution. Science. 2013;342:976–9. doi: 10.1126/science.1244730 24264991 10.1126/science.1244730

[35] Wiley D, Wilson IA, Skehel JJ. Structural identification of the antibody-binding sites of Hong Kong influenza haemagglutinin and their involvement in antigenic variation. Nature. 1981;289:373–8. 6162101 10.1038/289373a0

[36] Kaverin N, Rudneva IA, Govorkova EA, Timofeeva TA, Shilov AA, Kochergin-Nikitsky KS, et al Epitope mapping of the hemagglutinin molecule of a highly pathogenic H5N1 influenza virus by using monoclonal antibodies. J Virol. 2007;81:12911–7. doi: 10.1128/JVI.01522-07 17881439 10.1128/JVI.01522-07 PMC2169086

[37] Nakajima S, Nakajima k, Nobusawa E, Zhao J, Tanaka S, Fukuzawa K. Comparison of epitope structures of H3HAs through protein modeling of influenza A virus hemagglutinin: mechanism for selection of antigenic variants in the presence of a monoclonal antibody. Microbiol Immunol. 2007;51:1179–87. 18094536 10.1111/j.1348-0421.2007.tb04013.x

[38] Skowronski D, Janjua NZ, De Serres G, Sabaiduc S, Eshaghi A, Dickinson JA, et al Low 2012–13 Influenza Vaccine Effectiveness Associated with Mutation in the Egg-Adapted H3N2 Vaccine Strain Not Antigenic Drift in Circulating Viruses. PLoS ONE. 2014;3:e92153. 10.1371/journal.pone.0092153 PMC3965421 24667168

[39] Sun S, Wang Q, Zhao F, Chen W, Li Z. Glycosylation Site Alteration in the Evolution of Influenza A (H1N1) Viruses. PLoS ONE. 2011;6:e22844 doi: 10.1371/journal.pone.0022844 21829533 10.1371/journal.pone.0022844 PMC3145772

[40] Air G, Els MC, Brown LE, Laver WG, Webster RG. Location of antigenic sites on the three-dimensional structure of the influenza N2 virus neuraminidase. Virology. 1985;145:237–48. 2411049 10.1016/0042-6822(85)90157-6

[41] Colman P, Varghese JN, Laver WG. Structure of the catalytic and antigenic sites in influenza virus neuraminidase. Nature. 1983;303:41–4. 6188957 10.1038/303041a0

[42] ECDC. Influenza virus characterisation, summary Europe, March 2016. 2016. Available from: http://ecdc.europa.eu/en/publications/Publications/influenza-virus-characterisation-june-2016.pdf.

[43] Ayora-Talavera G, Montalvo-Zurbia FG, Gomez-Carballo J, Gonzalez-Losa R, Conde-Ferraez L, Puerto-Solís M, et al Influenza seasonality goes south in the Yucatan Península: The case for a different influenza vaccine calendar in this Mexican region. Vaccine. 2017 http://dx.doi.org/10.1016/j.vaccine.2017.07.020 10.1016/j.vaccine.2017.07.020 28755836

