# Supplementary figures and images for: Temporal distribution and genetic variants in influenza A(H1N1)pdm09 virus circulating in Mexico, seasons 2012 and 2013
Source: PLoS One. 2017 Dec 8;12(12):e0189363. doi: 10.1371/journal.pone.0189363 (PMC5722308; doi:10.1371/journal.pone.0189363)

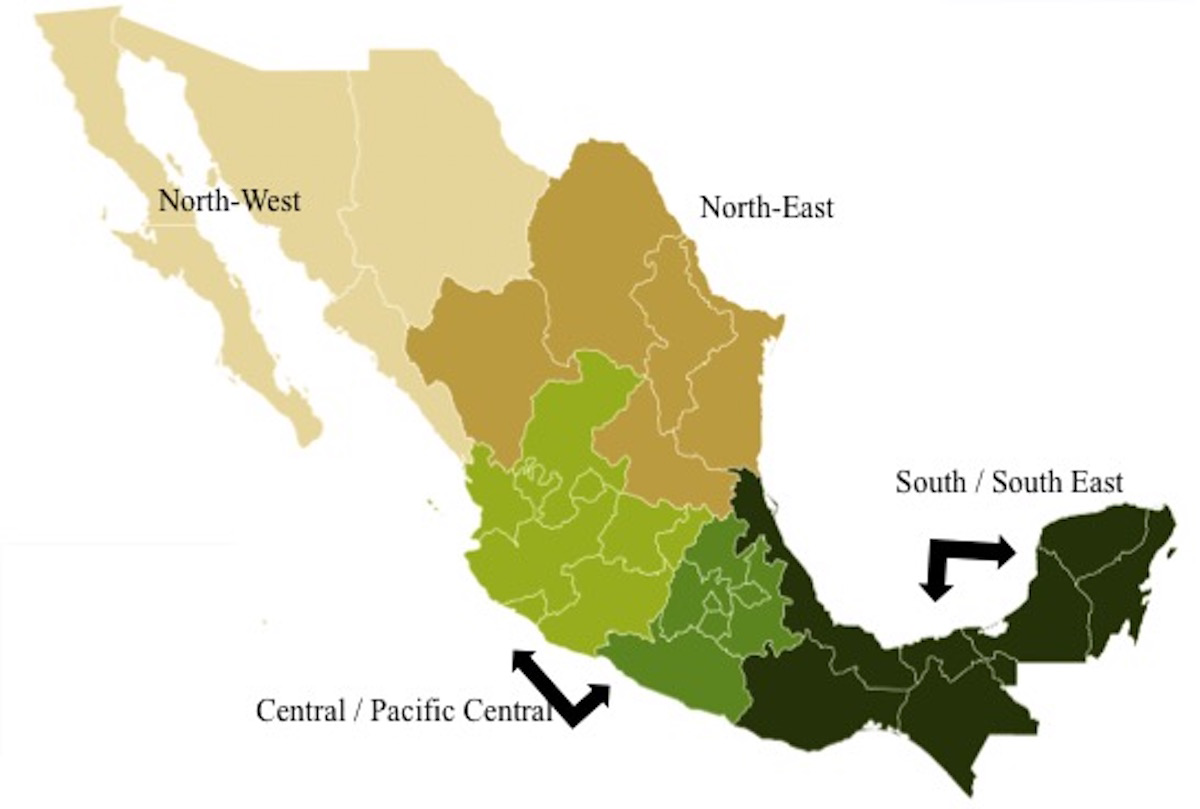

Supplement: S1 Fig — (TIF) [file pone.0189363.s002.tif]

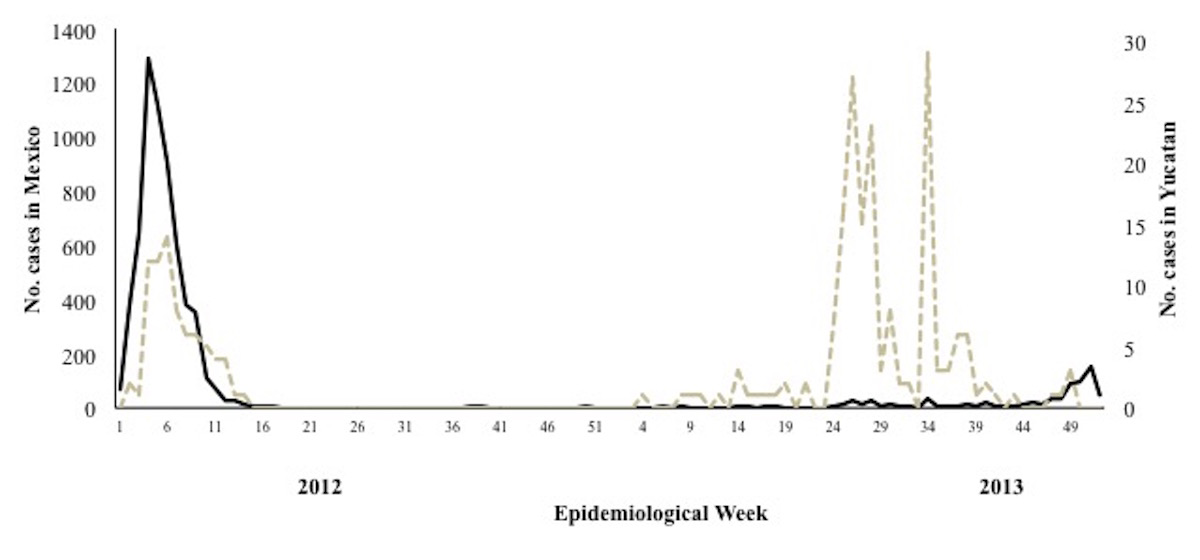

Supplement: S2 Fig — The black solid line represents number of confirmed cases by laboratory in Mexico including Yucatan (y axe). The dotted line represents number of confirmed cases by the Regional laboratory in Yucatan (secondary y axe). (TIF) [file pone.0189363.s003.tif]

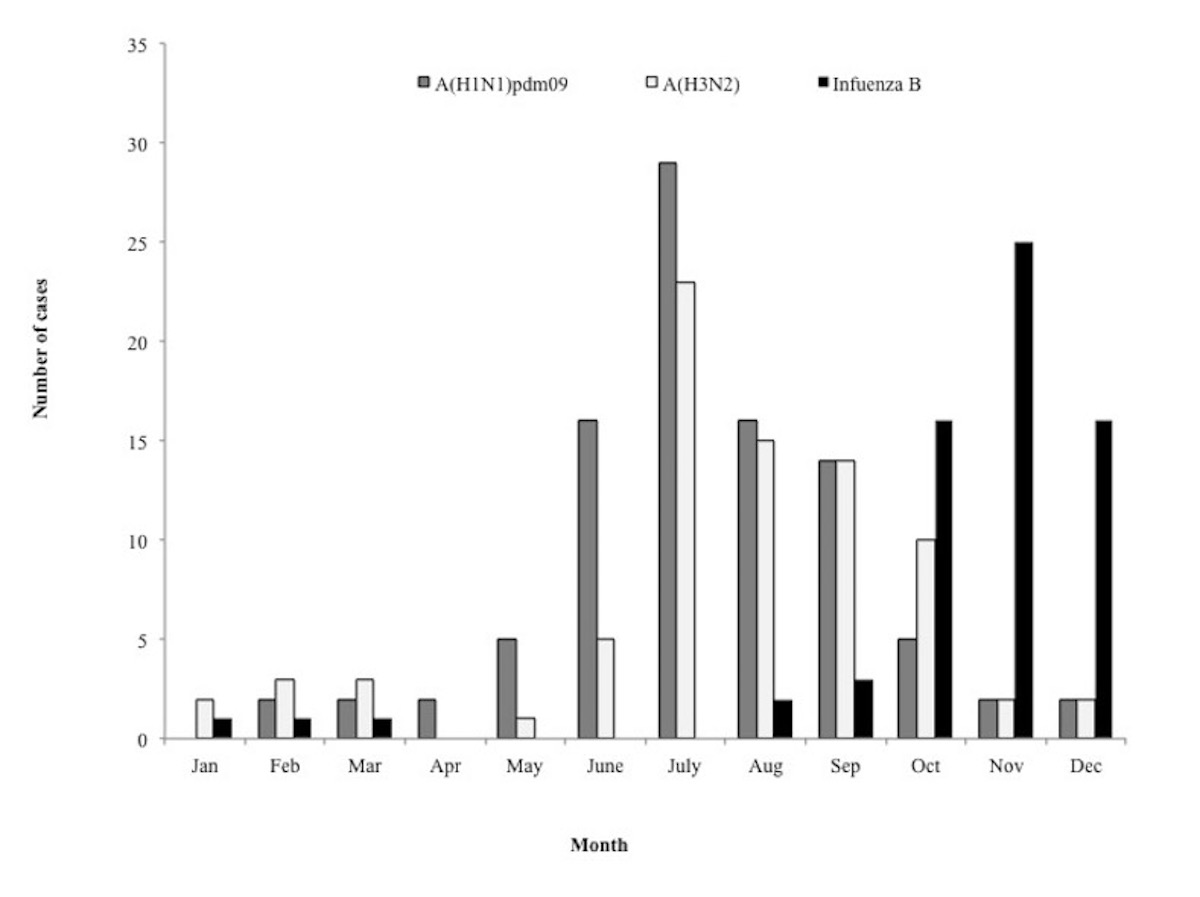

Supplement: S3 Fig — Influenza AH3N2 and A(H1N1)pdm09 were circulating at the same time from May until October. The A(H1N1)pdm09 peaked in circulation from June to October whereas the H3N2 peaked from July to October. At the end of the year, the influenza epidemic was predominated by influenza B virus. (TIF) [file pone.0189363.s004.tif]

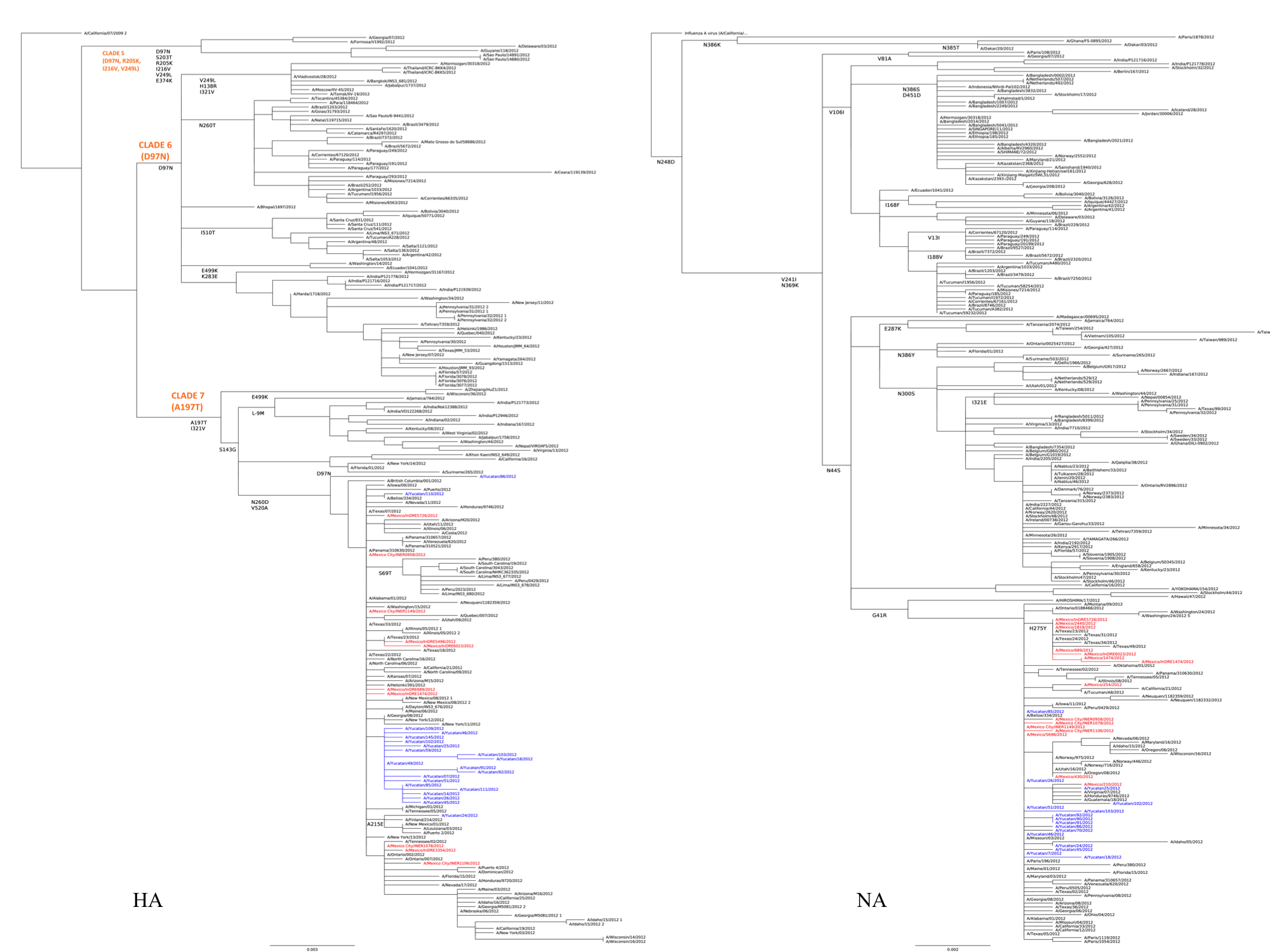

Supplement: S4 Fig — Trees were constructed for HA and NA full-length nucleotide sequences from different regions of the world. Viruses are coloured in black to represent worldwide strains, red for viruses from Mexico (non-Yucatan), and blue for viruses specifically from Yucatan. Trees were constructed using the PHYML method with a SH-like branch support. (TIFF) [file pone.0189363.s005.tiff]
